# Progress in Surgery

**Published:** 1899-06-17

**Authors:** 


### Progress in Surgery.

### SURGERY OF THE RECTUM AND ANUS.

The surgery of the rectum is in a progressive state, not only in treatment but in diagnosis. Martin (Cleveland) has called attention to the inexactitude of location as "so many inches from the anus." In reality the levator ani determines the position of the fixed and moveable rectum, the one uncovered and the other invested with peritoneum. The fixed rectum is of variable length under different conditions. Taking one of the fixed valves it is found to be capable of a range of movement of two to three inches. It is therefore obvious that some convenient topographical subdivision of the rectum is urgently needed, and perhaps this will be ultimately based on the relationship of the rectal valves.

A proctoscope of four inches length may be conveniently used, the rest of the rectum being inflated in the genu-pectoral position. A special chair has been introduced for putting the patient passively in such a position. The principle of inflation and inspection was introduced by Howard Kelly in 1895. Dr. James Tuttle speaks well of the method as applied to the rectum.[1] He figures a sigmoid cancer detected by the instrument some twelve inches from the anus. It is his opinion that morning diarrhœa and mucous diarrhœa are always the result of some actual disease of the lower bowel.

Wounds of the peritoneal portion of the rectum form the subject of a paper by M. Quénu.[2] They are chiefly caused by falls upon sharp instruments or by the introduction of rectal dilators, sounds, and the like. The liver, diapraghm, chest and other parts have been simultaneously wounded by the perforating instrument. Pain and collapse may be very severe from the first, or may be absent altogether. Septic peritonitis is the usual and fatal termination. Hence it is necessary in every case to verify by sight and touch the condition of the rectal walls. To do this a speculum should be passed, under anæsthesia if necessary. The wound should be carefully cleansed and then probed to determine if it has passed beyond the rectal wall. When perforation is discovered laparotomy ought to be performed as soon as possible, the patient being in the Trendelenburg position, and the viscera turned out into warm cloths. The prognosis, however, is bad, the mortality, even after operation, being over 30 per cent.

In the report of a clinical lecture by H. G. Howse,[3] he advocates very early incision into ischio-rectal abscesses, owing to a great tendency to gangrenous inflammation from the intensity of the process. An abscess due to an ulcer high in the rectum may form above the level of the levator ani.

Matthews, of Kentucky, in a paper on Rectal Surgery, gives some pointed remarks. He thinks all external hæmorrhoids thrombosed or not should be cut off,[4] and that ligature is the best operation for internal piles. He insists on the necessity of thoroughly slitting up every fistulous tract rather than merely dividing a fistula. He regards nearly all ulcerations of the rectum as tuberculous, syphilitic, or cancerous. Tuberculous ulcers he excises with the knife. Syphilitic fibrous strictures he treats by radical excision, for it is incurable by either local or general applications. He says this is, indeed, a melancholy class of patients as incurable as cancer, with the disadvantage over the latter that life is for much longer period made a thing of much misery and suffering. Cancer of the rectum the author beholds with horror when he contemplates the comparative futility of operation.

Benign new growths of the rectum are rare. Before a growth be pronounced non-malignant it should be histologically examined. The simple new growths which appear to be authenticated are myxomata (?), enchondromata (?), fibromata, lipomata, myomata, and fibromyomata. Over a certain size the pedunculated growths of the rectum can only be of connective tissue origin. The complex connective tissue tumours are angiomata, teratomata, and dermoid cysts.[5]

Bodenhamer points out that the polypi which occur in the rectum are also common to the mucous cavities of the uterus, vagina, nose, bladder, &c. They occur in various sites, and are usually single. Occasionally, however, they are very numerous and small, giving rise to the name granuloma of the rectum. The author notes the debilitating effect of the mucoid discharge from villous papillomata. It must ever be remembered that polypoid tumours of the rectum may bring down with them a pouch of peritoneum.

The advantages of rest to the colon in chronic membranous colitis, advocated by the late Greig Smith and others, have been well shown in a case described by Dr. J. M. Lawrie. In this case a colotomy opening was made for seven months, and enabled the patient to recover.[6]

The most recent advances in the cure of hæmorrhoids are in the direction of injection and of excision. Several surgeons advocate and practise isolated excision of the individual piles, followed by suture of the cut mucous membrane. Byford (Chicago)[7] and Thelwall Thomas[8] have recently written on the subject; the latter uses a continuous reversing suture with needle at each end. Schiff[9] does a similar operation.

A new method of excision of the rectum for cancer has been described by Carwardine.[10] The case operated on was one of spindle-celled sarcoma of the rectum, probably the first published in Great Britain. Mr. Carwardine places the patient prone, with the buttocks raised and the legs hanging over the end of the table. If an extensive and aseptic operation is desired a preliminary colotomy may be performed some days beforehand. The coccyx is first excised subperiosteally. The sphincter is then stretched and a sharp knife passed through the anus and coccygeal periosteum, dividing everything backwards in the middle line. The lower part of the sacrum should also be removed as well, as a rule. The whole of the bowel is divided except above where the mucous membrane is alone severed, and by this means the presacral tissue, containing any enlarged glands, can be dissected up in continuity, and the peritoneum be deflected from it. It is then divided high up in the hollow of the sacrum. The mucous membrane of the sphincter is left, and the upper part of the bowel united to the lower, and the wound almost completely closed. The great advantages of this operation are the freedom of exposure of the bowel and the facilities for treating difficult cases, the preservation of the sphincter ani with its nerve supply, and of the rectal sense, and the renewal of the bony coccyx from the periosteum. Patients treated by operation in this way are able subsequently to completely control their motions and flatus, and have the natural condition of the parts restored, contrasting with the lamentable state of the patient after such an operation as Kraske's.

Keen recommends a modification of Kraske's operation, stating that it is advantageous to completely close the rectum and leave a permanent abdominal anus.[11] Probably, the procedure of M. Quénu would be preferable to this. He has successfully extirpated the whole rectum in two cases with complete success, leaving a permanent anus in the loin.[12]

Pruritus ani is a troublesome affection having many causes and often resisting many remedies. Mr. F. C. Wallis believes that it is often the consequence of an ulcer of the rectum. He finds that in at least 25 per cent. of cases there is between the external and internal sphincters a small superficial ulcer situated posteriorly. He regards it as the most common cause of the condition.[13]

[1] New York Med. Jour., July 2, 1898. [2] Rev. de Chir., Jan., 1899. [3] Indian Med. Rec., Sept. 1, 1898. [4] Inter. Med. Mag., July, 1898. [5] Longuet le Progrès Médicale, Aug. 27, 1898. [6] Brit. Med. Jour., Nov. 5, 1898. [7] Annals of Surgery, Mar., 1899. [8] Brit. Med. Jour., Nov. 26, 1898. [9] Med. Rec., Dec. 31, 1898. [10] Brit. Med. Jour., Dec. 17, 1898. [11] Brit. Med. Jour., Mar. 11, 1899. [12] Rev. de Chir., Aug., 1898. [13] Clin. Jour., Jan. 11, 1899.

